# A barcoding‐based scat‐analysis assessment of Eurasian otter *Lutra lutra* diet on Kinmen Island

**DOI:** 10.1002/ece3.7712

**Published:** 2021-06-04

**Authors:** Nian‐Hong Jang‐Liaw

**Affiliations:** ^1^ Conservation Genetics Laboratory Conservation and Research Center Taipei Zoo Taipei City Taiwan

**Keywords:** diet, DNA barcode, Kinmen Island, *Lutra lutra*, Sanger sequencing

## Abstract

1. While it is well known that Eurasian otters principally feed on fishes and crustaceans, their detailed diet taxonomies are not fully understood. This is partly due to their nocturnal behavior and the limited resolving power of traditional morphological identification from scat. A suitable, reliable molecular method for diet studies is therefore needed.
2. I performed a series of Sanger‐sequencing reactions, utilizing nine primer sets for Eurasian otter diet research. These are mainly based on the barcoding concept to determine the taxonomic composition of spraints. The primer sets target different types of animals, amplifying each separately. This procedure was used to detect the prey contents of 64 spraint samples collected from Kinmen Island. Through high‐resolution gel electrophoresis and sequencing, it was evident that PCR products could be successfully amplified by the different primer sets and from spraint samples comprising multiple prey species.
3. Extracted DNA from all spraint samples was PCR‐amplified with 9 primer sets. In total, 16 prey types were identified across all 64 samples. Fourteen were identified at the species level.
4. The aim of this study was to develop and apply a novel diet research method to Eurasian otters. Eight of the primers are universal primers designed for COI segments of different animal groups, and one primer set was designed specifically for tilapia groups. This method can be applied to study the diets of not only Kinmen Eurasian otter populations, but also other Eurasian otter populations and other small carnivorous animals.

## Graphical abstract

A Eurasian otter eating a tilapia in Tai Lake, Kinmen. The photograph was taken by Fu‐Sheng Huang in June 2020.

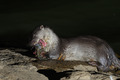

## 1 INTRODUCTION

**FIGURE 1:**
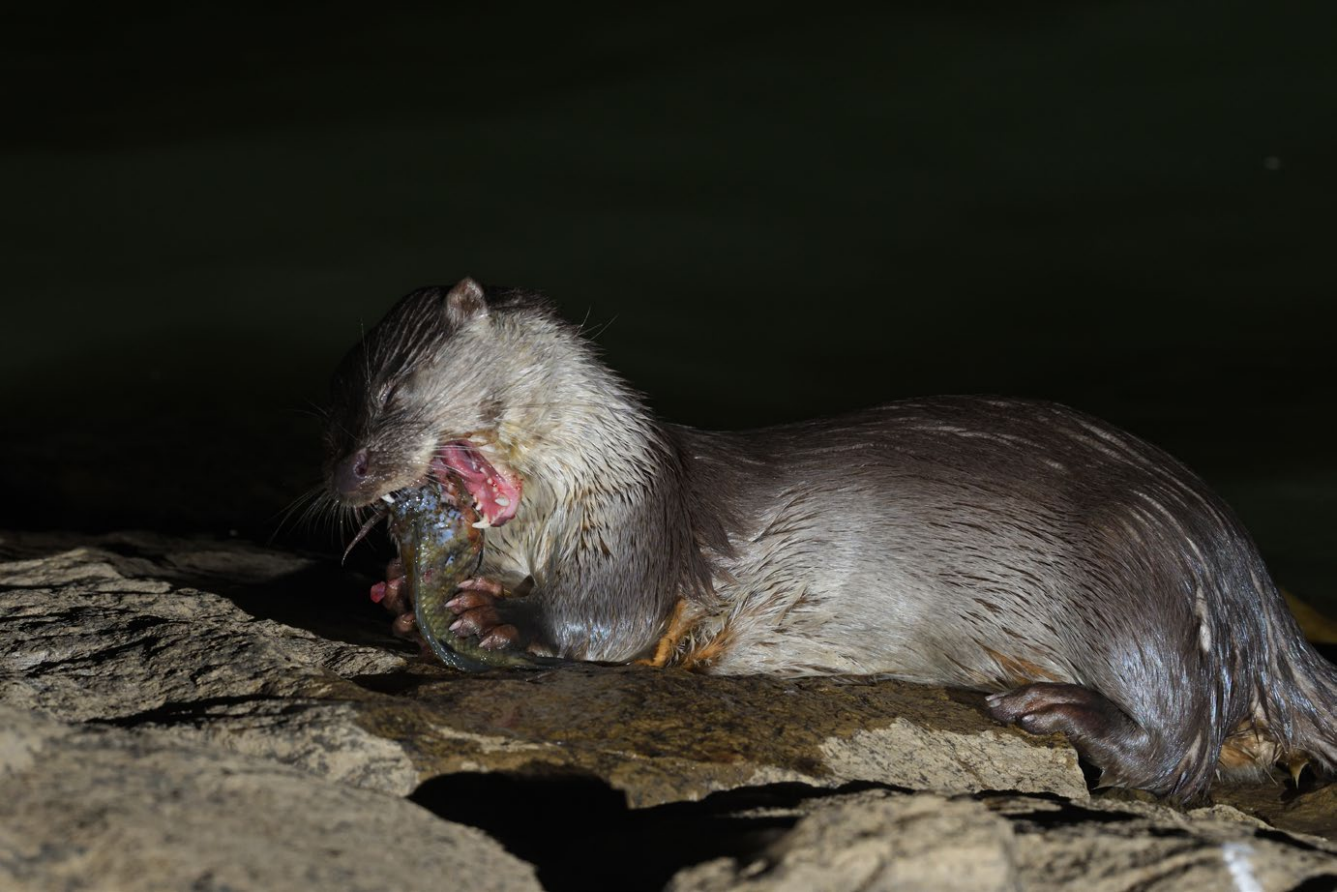
A Eurasian otter eating a tilapia in Tai Lake, Kinmen (site 11 of this study). The photograph was taken by Fu‐Sheng Huang in June 2020

The Eurasian otter *Lutra lutra* is a semi‐aquatic carnivore belonging to the Mustelid family. In many areas of Asia, Eurasian otters remain a species of conservation concern following widespread population declines during the 20th century (Li & Chan, 2018; Zhang et al., 2016). Currently, Kinmen Island maintains the most stable Eurasian otter populations in South‐East Asia (Figure 1; Hung et al., 2004; Lee, 1996). Compared to nearby areas in China, this small island provides a refuge for Eurasian otters and other wildlife, due to its battlefront position in the Taiwan Strait and strictly limited land use by local people in the decades prior to 1992 (You et al., 2013). Though some surveys of Kinmen's otter population structure and dynamics have been performed (Lee, 1996), ecological data on this population are otherwise very limited.

Diet analysis is a precondition to understanding the biology of a species and their interactions with others, as well as the functioning of ecosystems. Such studies therefore provide important data for understanding animal ecology, evolution, and conservation (Buglione et al., 2020; Jedlicka et al., 2016; Krahn et al., 2007; Rolfe et al., 2014; Shehzad et al., 2012; Symondson, 2002; Tournayre et al., 2021; Zhong et al., 2019). In prior studies, diets were mainly determined by direct observation of feeding, or by microscopic examination of gut contents or feces. Such traditional diet analyses have provided an abundance of useful data (Almeida et al., 2012; Carss, 1995; Heggberget & Moseid, 1994; Liu et al., 2018; Pierce & Boyle, 1991; Wasser et al., 1997). Nevertheless, they also have known biases and limitations. Direct observation approaches preclude working on tiny animals, most nocturnal species, anything beneath the soil, under water, hidden or elusive, while microscopic examination is labor‐intensive and relies on the researchers’ skill in identifying species from masticated, semidigested pieces of food (Liu et al., 2018; Moreby, 1988; Pierce & Boyle, 1991). Most of all, identification at the species level is difficult to achieve with these traditional diagnostic approaches (Carss, 1995). An accurate technique for determining the taxonomic composition of a species’ diet is therefore greatly needed.

When prey are too thoroughly digested for recognition, or when food species cannot otherwise be diagnosed from fecal remains (mollusks without bones, for example, or part of individuals such as soft muscle tissue), molecular identification of prey may be the only practical means of procuring data on trophic interactions that are difficult—if not impossible—to obtain in any other way (Liu et al., 2018; Symondson, 2002). Consequently, there is potential for applying such molecular approaches—and specifically, following the DNA barcoding concept—for otter diet analyses (Marcolin et al., 2020). However, prey DNA in feces is often highly degraded, preventing the amplification of long fragments for analysis (Lanszki & Molnár, 2003; Sittenthaler et al., 2019; Wasser et al., 1997). In early molecular studies, most attempts to analyze diet were performed by cloning PCR products and through subsequent Sanger sequencing of these clones by capillary electrophoresis (Deagle et al., 2005, 2007; Guillaud et al., 2017; Jarman et al., 2004; Valentini et al., 2009). These approaches are both time‐consuming and expensive (Pegard et al., 2009; Shehzad et al., 2012). Notably, Hong et al. (2019) used a Sanger sequencing‐based approach to identify vertebrate species from individual bones isolated from otters’ feces (spraints). This approach is laborious and requires technical expertise that limits the capacity of data generation, ignoring all information from boneless food items.

At present, next‐generation sequencing (NGS)‐based diet analysis of complex DNA mixtures such as feces, for example, scat DNA metabarcoding (sDNA metabarcoding), is becoming increasingly useful. This approach facilitates the generation of abundant sequence data from very large numbers of individual DNA molecules, deriving from a complex mixture and without the need for cloning (Schuster, 2008; Valentini et al., 2009). Such sDNA metabarcoding has already been applied to study several animal species from highly diverse taxa, as well as Eurasian otters (Buglione et al., 2020; Kumari et al., 2019; Pertoldi et al., 2021).

Nevertheless, there are several limitations to DNA metabarcoding‐based diet analyses of Eurasian otters. First, NGS is still prohibitively expensive for many smaller labs. Second, NGS data analysis can be time‐consuming and requires special knowledge of bioinformatics to garner accurate information from sequence data (Grada & Weinbrecht, 2013). Third, while the sensitivity of NGS is vastly superior to Sanger sequencing and is capable of detecting very low DNA concentrations, this high sensitivity is a double‐edged sword: It also facilitates the amplification of minute quantities of contaminating DNA (King et al., 2008), as well as secondary prey. Such organisms are potentially ingested by and/or attached to larger organisms predated by the otters, else were eaten by predatory fishes or other animals that contained them in their guts. The huge sequence output of NGS will thus include a high number of species derived from contamination or secondary predation, confusing our understanding of the real predation behavior of these otters.

Here, I present a barcoding‐based spraint‐analysis procedure, which I use to assess the diet of the Eurasian otter on Kinmen Island. This approach is based on Sanger sequencing with 9 primer sets, each targeted toward different prey taxa, and thus allowing for greater resolution than morphological studies. The aim of this work was to (a) provide an easy and affordable molecular method for detecting the species contained in spraint samples of Eurasian otter on Kinmen; (b) to demonstrate the performance of each of the primer sets; (c) to develop an efficient, custom‐designed primer set for the most common prey species group, tilapia, of otters in Kinmen; (d) to discuss the results of the sequencing and the limitations of this method, where applicable; and (e) to provide new best practices for studying the diets of Eurasian otters and those of other obligate carnivores that feed on similar prey.

## 2 MATERIALS AND METHODS

### 2.1 Study area

**FIGURE 2:**
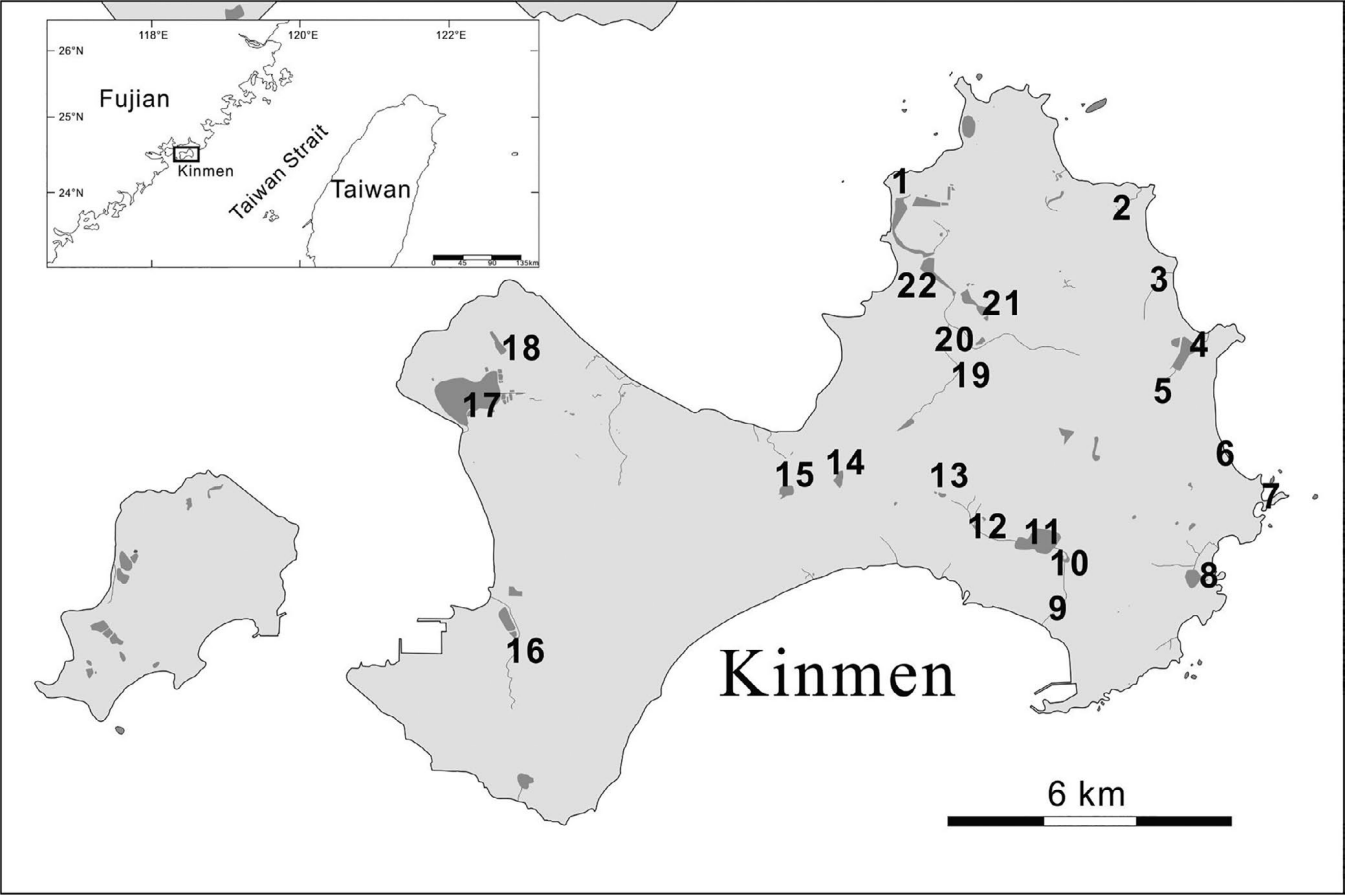
Locations of sampling sites in this study. Detailed location information is listed in Table 1

Kinmen Island is located 10 km (6.2 mi) off the southeastern coast of mainland China. It was originally a military reserve and a frequent battlefront between 1949 and 1979, before it was returned to the civilian government in the mid‐1990s. For agricultural and military needs, many reservoirs, artificial lakes, and ponds were constructed for storing water, raising fish, and irrigation on Kinmen. My colleagues and I collected spraint samples from 22 sites on Kinmen from April 2017 to November 2018. These collection sites can be catalogued as 6 types: freshwater stream (7 sites), freshwater pond (5), freshwater and brackish reservoirs (5 and 1, respectively), rocky coast (2), sand beach (1), and brackish wetland (1) (Figure 2).

### 2.2 Spraint samples used in this work

Scat freshness affects the proportion of detectable food DNA (McInnes et al., 2017). In this study, I used the freshest spraint samples (including jelly‐like and mucosal spraints) as possible. Furthermore, all spraint samples used in this work passed the DNA prescreening quality control procedure suggested by Hung et al. (2004) with few modifications. This procedure was applied to check the qualities of extracted DNA for subsequent individual identification procedures using microsatellite methods. Though such tests are time‐consuming, they facilitate exclusion of poor‐quality spraints and minimize the occurrence of false negatives in diet analysis.

Finally, 64 “very fresh” spraint samples, as catalogued by Lerone et al. (2014) were collected. Some samples contained special materials beside fish remains, such as hairs or feathers, broken shells, and bird bones which were observed by eye or via microscope prior to DNA extraction, as recorded in the “Remark” column (see Table 1). Fresh spraints were collected and preserved in 99% alcohol individually and kept frozen at −80°C until examination.

**TABLE 1:** Sample information and results of barcoding identification

Locality		Sample		Habitat types	Collection date	Primer set									Food species no.	Nonfood species	Valid sequence no.	Remark
#	Name	#	Code			I	II	III	IV	V	VI	VII	VIII	IX				
1	Xiyuan Coast	#1	XYH‐1	Rocky shore/S	20‐Nov‐17	–	–	–	N^a^	–	N^b^	N^c^	–	–	0	Shewanella algae*^a^, *Vibrio parahaemolyticus* ^b^, *V. diabolicus* ^c^	3	Dry; few; boneless
2	Shanhou	#2	SH‐20	Pond/F	19‐Nov‐17	N^LL^	MLa*	–	–	MLa*	N^LL^	–	–	N^LL^	1	*Lutra lutra* ^LL^	5	
		#3	SH‐21	Pond/F	19‐Nov‐17	–	–	–	N^LL^	N^a^	N^LL^	N^b^	–	–	0	*Lutra lutra* ^LL^, *Shewanella bicestrii* ^a^, Macrotrachela quadricornifera**^b^	4	Few
		#4	SH‐23	Pond/F	19‐Nov‐17	–	–	–	–	Nil	Nil	N^a^	N^b^	Nil	1	Uncultured bdelloid rotifer**^a^, Mosquitofish^b^	5	Few
		#5	SH‐27	Pond/F	19‐Nov‐17	Nil	–	–	N^LL^	Nil	Nil	–	–	Nil	1	*Lutra lutra* ^LL^	5	
3	Hobien Stream	#6	HB‐3	Stream/F	20‐Nov‐17	–	–	–	–	–	Nil	MN	–	Zi	3		3	Broken crab shell
4	Tianpu Reservoir	#7	TP‐76	Reservoir/F	8‐Aug‐17	–	–	–	N^LL^	CC	–	–	–	–	1	*Lutra lutra* ^LL^	2	Hairs
		#8	TP‐77	Reservoir/F	8‐Aug‐17	–	–	–	–	Bird	–	–	N	–	1	Mosquitofish	2	Toe's bone with skin
		#9	TP‐78	Reservoir/F	8‐Aug‐17	–	–	–	–	Bird	–	–	–	N^LL^	1	*Lutra lutra* ^LL^	2	Feathers
		#10	TP‐85	Reservoir/F	9‐Aug‐17	–	–	–	–	–	Zi	N	–	Zi	1	*Cladotanytarsus gracilistylus*	3	
5	Qianpu R.	#11	QP‐79	Stream/F	9‐Aug‐17	–	–	–	N^LL^	–	Bird	–	–	–	1	*Lutra lutra* ^LL^	2	Wet
		#12	QP‐81	Stream/F	9‐Aug‐17	–	–	–	–	–	Zi	–	–	Zi	1		2	
		#13	QP‐87	Stream/F	9‐Aug‐17	–	CC	–	–	–	Zi	–	–	Zi	2		3	
6	Goyu Bay	#14	FG‐13	Sand beach/S	20‐Nov‐17	–	–	Nil*	Nil	–	N^a^	–	N^b^	–	1	*Microphilypnus* sp.**^a^, Ant^b^	4	
7	Fuguodun Coast	#15	FG‐14	Rocky shore/S	20‐Nov‐17	–	MLs	PJ*	–	MLs*	–	N^a^	N^b^	MLa*	3	Awacaris yezoensis**^a^, *Garrha* sp.*^b^	6	
8	Jinhu Reservoir	#16	JH‐37	Reservoir/B	20‐Nov‐17	–	–	–	–	–	–	–	–	Zi	1		1	
		#17	JH‐38	Reservoir/B	20‐Nov‐17	–	–	–	–	–	N^LL^	–	–	–	0	*Lutra lutra* ^LL^	1	Black mucus
		#18	JH‐39	Reservoir/B	20‐Nov‐17	–	–	–	N^LL^	–	N^LL^	–	–	Nil	1	*Lutra lutra* ^LL^	3	
9	Bailong River	#19	BL‐8	Stream/F	18‐Nov‐17	–	–	–	–	–	–	–	–	Zi	1		1	Black mucus
10	Huanglong Lake	#20	HL‐24	Pond/F	18‐Apr‐17	N^LL^	No match	–	N^LL^	–	N^LL^	–	–	–	0	*Lutra lutra* ^LL^	4	Soft wet; boneless; very fresh (cub?)
		#21	HL‐39	Pond/F	8‐Aug‐17	–	Zi*	–	–	–	Zi	–	–	Zi	1		3	
		#22	HL45	Pond/F	18‐Nov‐17	–	–	–	N^LL^	Zi	Zi	N^a^	–	Zi	1	Lutra lutra*^LL^ *, Brachionus* sp.^a^	5	
11	Tai Lake	#23	Tai‐117	Reservoir/F	21‐Apr‐17	–	–	–	–	–	–	–	–	Zi	1		1	Greenish; soft
		#24	Tai‐120	Reservoir/F	21‐Apr‐17	–	–	–	–	–	–	–	–	–	0		0	Black mucus
		#25	Tai‐127	Reservoir/F	21‐Apr‐17	–	MN	–	–	–	N	–	–	–	1	Shewanella oneidensis*	2	Browndish; soft; segmental appendages
		#26	Tai‐131	Reservoir/F	8‐Aug‐17	–	–	–	–	–	Zi	–	–	–	1		1	
		#27	Tai‐134	Reservoir/F	8‐Aug‐17	–	–	–	–	Nil	Nil	–	–	Zi	2		3	
		#28	Tai‐140	Reservoir/F	18‐Nov‐17	–	–	–	–	Zi	Zi	–	–	–	1		2	
		#29	Tai‐141	Reservoir/F	18‐Nov‐17	–	–	–	–	–	Zi	–	–	–	1		1	
		#30	Tai‐173	Reservoir/F	30‐May‐18	–	–	–	–	–	–	–	–	–	0		0	Few; soft wet; boneless (cub?)
		#31	Tai‐174	Reservoir/F	30‐May‐18	–	–	–	–	–	–	–	–	–	0		0	Yellow; few; soft wet; boneless (cub?)
12	Shanwai Stream	#32	YB‐20	Stream/F	23‐Apr‐17	–	–	–	–	–	Zi	MN	–	–	2		2	
		#33	YB‐25	Stream/F	23‐Apr‐17	–	–	–	–	–	–	–	–	Zi	1		1	
		#34	YB‐59	Stream/F	8‐Aug‐17	–	–	–	–	Zi	Zi	–	N	Zi	1	Cyclotella cryptica*	4	
		#35	YB‐61	Stream/F	8‐Aug‐17	–	–	–	–	–	Zi	–	–	–	1		1	
		#36	YB‐70	Stream/F	18‐Nov‐17	–	–	–	–	–	–	–	–	–	0		0	
		#37	YB‐72	Stream/F	18‐Nov‐17	–	–	–	–	–	–	–	–	–	0		0	Greenish; small
		#38	YB‐75	Stream/F	17‐Nov‐17	–	–	–	–	–	Zi	–	–	–	1		1	
13	Mintan Lake	#39	MT‐16	Pond/F	7‐Aug‐17	N^LL^	–	–	N^LL^	–	N^LL^	–	–	–	0	*Lutra lutra* ^LL^	3	Black mucus
		#40	MT‐19	Pond/F	7‐Aug‐17	–	–	–	–	–	–	–	N	Zi	1	*Philodina megalotrocha*	2	
		#41	MT‐20	Pond/F	7‐Aug‐17	–	–	–	–	–	–	–	–	Zi	1		1	
14	Lan Lake	#42	LAN‐71	Reservoir/F	18‐Apr‐17	–	–	–	N^LL^	–	N^LL^	–	–	Nil	1	*Lutra lutra* ^LL^	3	Greenish; soft
		#43	LAN‐73	Reservoir/F	18‐Apr‐17	–	–	–	–	–	N^LL^	–	–	Nil	1	*Lutra lutra* ^LL^	2	Greenish jelly
		#44	LAN‐74	Reservoir/F	18‐Apr‐17	–	N^a^	–	–	–	N^LL^	–	–	–	0	*Shewanella* sp^a^., *Lutra lutra* ^LL^	2	Black mucus
15	Qionglin Reservoir	#45	QL‐41	Reservoir/F	18‐Nov‐17	–	–	–	N^LL^	No match	–	–	–	–	0	*Lutra lutra* ^LL^	2	Greenish jelly
		#46	QL‐48	Reservoir/F	18‐Nov‐17	–	MN	Zi	Zi	MN	Zi	MN	MN	–	2		7	Greenish soft
		#47	QL‐53	Reservoir/F	18‐Nov‐17	–	–	–	–	–	–	–	–	–	0		0	Black mucus
16	Xianju	#48	SG‐1	Stream/F	20‐Nov‐17	–	–	–	–	–	Nil	–	–	–	1		1	
17	Ci Lake	#49	Ci‐18	Wetland/B	10‐Aug‐17	–	N	–	–	N	–	–	–	Zi	1	Idiomarinaceae bacterium**	3	
		#50	Ci‐38	Wetland/B	1‐Jun‐18	–	Snake	–	Snake	–	–	–	–	–	1		2	Snake skin
		#51	Ci‐44	Wetland/B	1‐Jun‐18	–	Snake	–	Snake	Snake	–	N	–	–	1	*Acanthamoeba* sp.*	4	Snake skin
18	Shuangli Lake	#52	SL‐18	Pond/F	23‐Apr‐17	–	–	–	–	–	N	–	–	Zi	1	Shewanella oneidensis*	2	
		#53	SL‐31	Pond/F	19‐Nov‐17	–	–	–	–	–	Mo	–	–	Nil	2		2	
19	Guangqian River	#54	GQR‐37	Stream/F	6‐Aug‐17	–	N	–	N	N	–	Crab	–	–	1	Shewanella bicestrii*	4	Reddish; segmental appendages
		#55	GQR‐39	Stream/F	6‐Aug‐17	–	–	Crab	–	–	–	Crab	–	Zi	2		3	Broken crab shell
		#56	GQR‐44	Stream/F	7‐Nov‐18	–	CC	–	–	CP	–	–	–	–	2		2	
20	Doumen River	#57	DMR‐65	Stream/F	6‐Aug‐17	–	–	–	N^a^	N^b^	–	Crab	–	–	1	Shewanella bicestrii*^a^, *Shewanella* sp.**^b^	3	Reddish; broken crab shell
		#58	DMR‐68	Stream/F	6‐Aug‐17	–	N^a^	–	N^a^	N^b^	N^c^	–	Crab	–	1	Shewanella bicestrii*^a^, *S. decolorationis* ^b^, *Aeromonas diversa* ^c^	5	Reddish
21	Rong Lake	#59	Rong‐51	Reservoir/F	7‐Aug‐17	–	–	–	–	Zi*	–	–	N	Zi	1	Cladotanytarsus gracilistylus*	3	
		#60	Rong‐55	Reservoir/F	17‐Nov‐17	–	–	–	–	–	–	–	–	–	0		0	Greenish jelly
		#61	Rong‐57	Reservoir/F	17‐Nov‐17	–	Zi	CM	N^LL^	–	CM*	–	–	Zi	2	*Lutra lutra* ^LL^	5	
22	Yangshan	#62	YS‐53	Pond/F	19‐Apr‐17	–	–	–	–	–	–	–	–	Zi	1		1	
		#63	YS‐54	Pond/F	19‐Apr‐17	–	–	–	–	N^a^	N^LL^	–	–	MJ	1	*Mugilogobius chulae^a^*, *Lutra lutra* ^LL^	3	
		#64	YS‐56	Pond/F	17‐Nov‐17	–	–	–	–	–	–	–	–	–	0		0	Brown jelly

Note

“Locality”: site of spraint collection. “Sample”: the codes of spraint samples. “Food Species no.”: diet species number of each spraint sample. “Valid sequence no.”: the number of all readable sequences from each sample. “Remark”: morphologic notes of unusual spraint samples.

The abbreviation codes of food species (in blue color) refer to Table 3, and superscript codes (a, b, c, LL) of nonfood species (in red color) refer to the “Nonfood species” column of this table for each sample. Primer set details refer to Table 2.

*: the highest similarity of sequence and compared data is higher 90% but less than 98%; ** is higher 80% but less than 90%. Species without star mark indicate that the similarity is higher than 98%.

### 2.3 DNA extraction

The DNeasy Blood & Tissue Extraction Kit (QIAGEN, Germany) was used according to the manufacturer's instructions, with few modifications as detailed in Appendix S1. The extracted DNA was suspended in 80 μl AE buffer.

### 2.4 Selection and design of primer sets

Each DNA sample extracted from spraints was PCR‐amplified 9 times and with 9 primer sets. I browsed the published universal COI primers and chose eight sets to estimate the diet contents of spraints collected in Kinmen. Besides the COI primers, a group of COIII primers was designed for this study for the most abundant prey species in Kinmen, the introduced tilapia. They are forward primer Til9020F and reverse primer cocktails Mos9516R+Nil9464R+Esc9305R+Zil9212R (TilMR). The details of primers used are listed in Table 2.

**TABLE 2:** PCR primer sets or cocktails used to amplify COI/COIII segments in this study

Set	Primer name/Cocktail name	Sequence 5′‐3′	Target gene	Target animals	Approx. sequence length (bp)	References
I	BirdF1	TTCTCCAACCACAAAGACATTGGCAC	COI	Birds	650	Hebert, Stoeckle, et al. (2004)
	BirdRM (mixed with BirdR1, R2 and R3)					
	BirdR1	ACGTGGGAGATAATTCCAAATCCTG				Hebert, Stoeckle, et al. (2004)
	BirdR2	ACTACATGTGAGATGATTCCGAATCCAG				Hebert, Stoeckle, et al. (2004)
	BirdR3	AGGAGTTTGCTAGTACGATGCC				Hebert, Stoeckle, et al. (2004)
II	VF1	TTCTCAACCAACCACAAAGACATTGG	COI	Mammals, reptiles, fish, amphibians, and some insects	650	Ivanova et al. (2006 )
	VRM (mixed with VR1 and VR1d)					
	VR1(FishR1)	TAGACTTCTGGGTGGCCAAAGAATCA				Ivanova et al. (2006)
	VR1d	TAGACTTCTGGGTGGCCRAARAAYCA				Ivanova et al. (2006)
III	chmf4	TYTCWACWAAYCAYAAAGAYATCGG	COI	Amphibians	650	Che et al. (2012)
	chmr4	ACYTCRGGRTGRCCRAARAATCA				Che et al. (2012)
IV	FF2d	TTCTCCACCAACCACAARGAYATYGG	COI	Fishes	650	Ivanova et al. (2007)
	FR1d	CACCTCAGGGTGTCCGAARAAYCARAA				Ivanova et al. (2007)
V	FishF1	TCAACCAACCACAAAGACATTGGCAC	COI	Fishes	650	Ward et al. (2005)
	FishR1	TCGACTAATCATAAAGATATCGGCAC				Ward et al. (2005)
VI	FishF2	TAGACTTCTGGGTGGCCAAAGAATCA	COI	Fishes	650	Ward et al. (2005)
	FishR2	ACTTCAGGGTGACCGAAGAATCAGAA				Ward et al. (2005)
VII	LCO1490	GGTCAACAAATCATAAAGATATTGG	COI	Various phyla from the animal kingdom	650	Folmer et al. (1994)
	HCO2198	TAAACTTCAGGGTGACCAAAAAATCA				Folmer et al. (1994)
VIII	LepF1	ATTCAACCAATCATAAAGATAT	COI	Lepidoptera	650	Hebert et al. (2004)
	LepR1	TAAACTTCTGGATGTCCAAAAA				Hebert et al. (2004)
IX	Til9020F	TAACAATRTACCAATGATGACGAG	COIII	Tilapia	See below	This study
	TilMR (mixed with Mos9516R, Nil9464R, Esc9305R and Zil9212R)					
	Mos9516R	ACCCAAAGTGATGTTCTGATG			548	This study
	Nil9464R	GCAGACGGCCAGGAAAGTAGAGC			500	This study
	Esc9305R	ATAGTTAGGGCGAGGGATTGAA			346	This study
	Zil9212R	AAGACGGCGGTGTTAAGCAGAGG			254	This study

The primer cocktails are more effective than conventional primers, facilitating barcode work on taxonomically diverse samples (Ivanova et al., 2007). In this study, the reverse primer cocktails demonstrate different strategies of PCR amplification. For COI gene primers, BirdF1/BirdRM and VF1/VRM, the reverse primers can anneal to various nucleotide types in same location of 3’ end of the sequence and amplify DNA segments in similar length even they are not from the same hosts’ DNA materials. Another cocktail primer set, Til9020F/TilMR for the COIII gene for tilapia, has four reverse primers, which amplify different lengths of sequenced DNA segments. I designed them to identify tilapia based on four common introduced tilapia species in Taiwan. The reference sequences for primer design were AY597335 in the GenBank database (*Oreochromis mossambicus*), GU238433 (*O. niloticus*), KM654981 (*O. esculentus*; Kinaro, Xue, Nyaundi, et al., 2016), and KM658974 (*Coptodon zillii*; Kinaro, Xue, Volatiana, 2016).

### 2.5 PCR amplification

The PCR was performed using a *Taq* polymerase master mix (PCR Master Mix; Hopegen, Taichung, Taiwan). Each PCR mixture (20 μl) contained 1 μl of the fecal DNA template and 0.4 μM of each primer, which means at least 9 μl of extracted fecal DNA was required for PCRs of all primer sets. The PCR thermal cycling conditions consisted of 5 min at 94°C, 40 cycles of 30 s at 94°C, 30 s at 50°C, 50 s at 72°C, and a final step of 5 min at 72°C, using a Biometra TRIO 48 Thermal Cycler (Analytik Jena, Jena, Germany).

### 2.6 Checking the PCR products with high resolution methods

**FIGURE 3:**
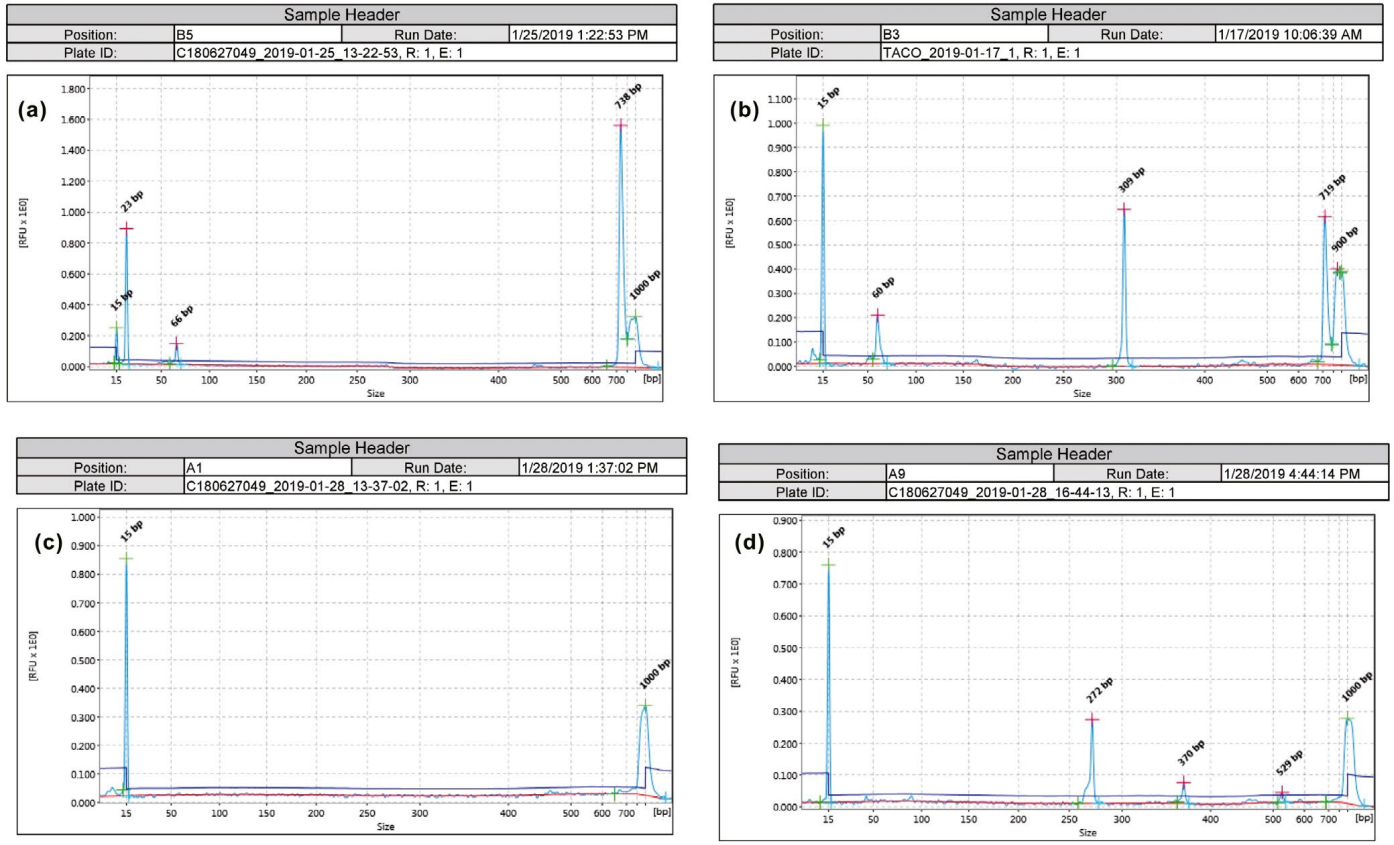
Examples of PCR results as inferred via the QIAxcel Advanced automated electrophoresis system. (a) a good PCR result of Tai‐131 (sample #26 in Table 1) with primers FishF2/R2, presenting a single peak. The smaller products (23 and 66 bp) should be dimer or fragment sequences and will be removed prior to sequencing; (b) a poor multiple‐peaks result of Lan‐74 (#44) with FishF1/R1, which implied several DNA segments were amplified in the PCR products; (c) a failed PCR of HB‐3 (#6) with chmf4/r4; no DNA was amplified; (d) another multiple‐peaks result of TP‐85 (#10) amplified with primer set Til9020F/RM; the PCR product was sequenced successfully

The Sanger method requires a single amplicon, comprising a single target, in order to produce a sequence. To ensure that PCR products were suitable for downstream sequencing, I ran each PCR product on the QIAxcel Advanced automated electrophoresis system (QIAGEN, Germany), which affords a resolution down to 3–5 bp. For the COI gene segments, I chose the PCR products with a single signal peak to sequence (Figure 3a). For the PCR products with primer set Til9020F/RM (COIII gene), multiple peaks are acceptable to sequence as they are caused by different 5’ end primers (see Figure 3d).

### 2.7 DNA sequencing

PCR products that clearly showed amplification of the appropriate number of base pairs (i.e., close to 700 bp) without multiple peaks (Figure 3) were purified using a clean‐up reagent (HT ExoSAP‐IT High Throughout PCR Product Cleanup Kit, Applied Biosystems) and Sanger‐sequenced on an Applied Biosystems 3730XL DNA Analyzer unidirectionally using the forward primers only. This protocol for the amplification of mtDNA was used on all samples of extracted fecal DNA.

### 2.8 Identification of sequences from spraint samples

Most sequences obtained from spraint samples, except those amplified with Til9020F/RM primers, were compared to reference sequences in the Barcode of Life Data System (BOLD) and GenBank to default to the species level if possible. Those from PCRs with Til9020F/RM primers (located on the CO III gene) were compared with reference sequences in GenBank only. I assigned the candidate preys’ sequences of DNA to species or higher taxonomic levels with a similarity lower than 98%, and no equivalent similarity to any other reference, following Clare et al. (2009). Each spraint containing a prey category was considered one “record” for that prey, regardless of the number of individuals present. Nontarget sequences, including bacteria, tiny insects or invertebrates, are ostensibly not the prey of Eurasian otters and thus will be identified and considered in the discussion.

## 3 RESULTS

### 3.1 Results from spraint samples

All 64 spraint samples were amplified with 9 primer sets. The results are listed in Table 1. In total, this yielded 576 PCR products, of which 153 (derived from 49 samples) were selected and sequenced successfully (i.e., a 26.56% success rate). A total of 15 samples showed no direct/possible food species inside them, though might still contain nonfood DNA sequences like bacteria, fungi, tiny invertebrates, and/or host genomic DNA. All valid sequence information is detailed in Appendix S2 and Appendix S3.

**FIGURE 4:**
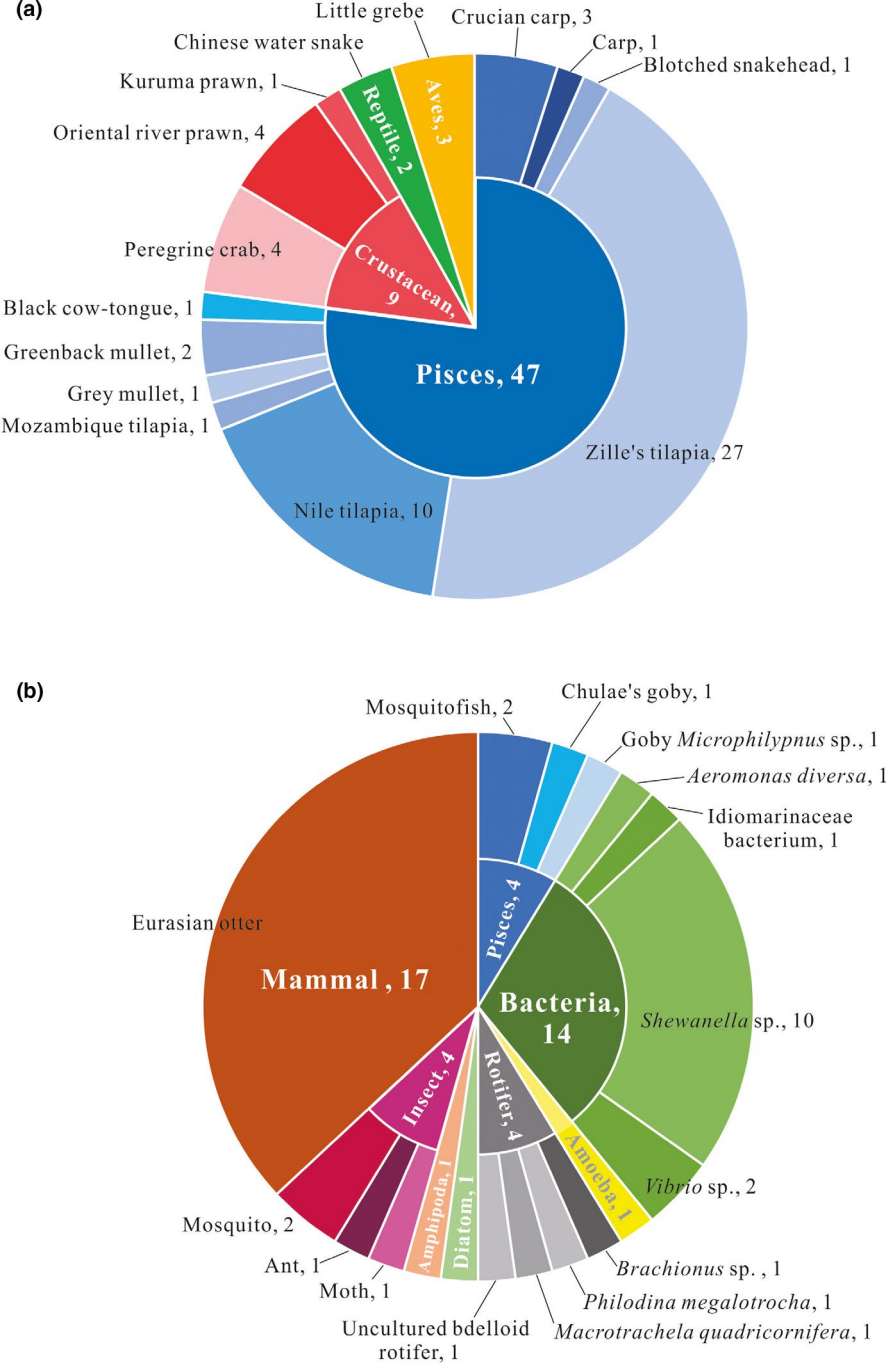
The food species (a) and nonfood species (b) sequenced in this study. Numbers following the species/catalogue are the repeat counts detected from the 64 tested spraint samples. More detailed information is recorded in Table 3

Based on sequencing of the 153 PCR amplicons, two kinds of fecal contents were identified. The first group comprised “food species” and thus is of relevance to diet research (Table 3a). A total of 39 fecal samples contained a single food species inside, 8 fecal samples contained two food species, and 2 fecal samples contained three food species. The most common food species was redbelly tilapia *Coptodon zillii* (Gervais, 1848), which was identified 27 times in 64 fecal samples, based on the barcoding identification of 41 PCR products; followed by Nile tilapia *Oreochromis niloticus* (Linnaeus, 1758) (10 counts), oriental river prawn *Macrobrachium nipponense* (De Haan, 1849) (4), peregrine crab *Varuna litterata* (Fabricius, 1798) (4), little grebe *Tachybaptus ruficollis* (Pallas, 1764) (3), crucian carp *Carassius auratus* (Linnaeus, 1758) (3), Chinese water snake *Enhydris chinensis* (Gray, 1842) (2), and mullet *Chelon* sp. (similar to *C. affinis* up to 97.54%, 2 counts), and Mozambique tilapia *O. mossambicus* (Peters, 1852), tonguefish *Paraplagusia* sp. (with similarity of 95.72% to Black cow‐tongue *P. japonica*), greenback mullet *Planiliza subviridis* (Valenciennes, 1836), carp *Cyprinus carpio* Linnaeus, 1758, blotched snakehead *Channa maculata* (Lacepède, 1801), and kuruma shrimp *Marsupenaeus japonicus* (Bate, 1888), each with one count. Two samples contained two tilapia species (Figure 4). In total, 3 tilapia species were identified in these spraint samples. They were *Coptodon zillii* (41 readable sequences from 28 samples), *Oreochromis niloticus* (17/11), and *O. mossambicus* (1/1). Tilapia as prey occurred in almost all habitats but the rocky coast (site 7) and comprised the most abundant prey species recorded; 54.69% spraint samples (35 of all 64 tested samples) contained tilapia sequences as food species. In total, 60.94% spraint samples (39 of 64 samples) contained fish as food species. Only 11 samples contained no fish species as food inside, excluding those that contained no food species at all. Few other fish species were recorded as prey and in only 5 samples (7.81% of total 64 samples). This was followed by crustaceans, present in 14.06% of samples (9/64), a single bird species (the little grebe *Tachybaptus ruficollis*) in 4.69% (3 samples) and an aquatic snake (*Enhydris chinensis*) in 3.13% (2 samples). No amphibians or mammals were found in this study as food species.

**TABLE 3:** Food species list (a)/Nonfood species list (b), repeat counts and percentage relative frequency of occurrence of various species detected from spraint samples of this study

	Habitat type														Total (64)	
	Freshwater (55)						Brackish water (6)				Sea (3)					
	Stream		Pond		Reservoir		Reservoir		Wetland		Sand beach		Rocky coast		No.	%
	No.	%	No.	%	No.	%	No.	%	No.	%	No.	%	No.	%		
**a. Food species**																
Pisces																
Crucian carp *Carassius auratus* (CC)	2	3.64			1	1.82									3	4.69
Carp *Cyprinus carpio* (CP)	1	1.82													1	1.56
Blotched snakehead *Channa maculata* (CM)					1	1.82									1	1.56
Zille's tilapia *Coptodon zillii* (Zi)	10	18.18	6	10.91	9	16.36	1	16.67	1	16.67					27	42.19
Nile tilapia *Oreochromis niloticus* (Nil)	2	3.64	3	5.45	3	5.45	1	16.67			1	33.33			10	15.63
Mozambique tilapia *O. mossambicus* (Mo)			1	1.82											1	1.56
Gray mullet *Chelon affinis* ^a^ (MLa)													1	33.33	1	1.56
Greenback mullet *Planiliza subviridis* ^a^ (MLs)			1	1.82									1	33.33	2	3.13
Black cow‐tongue *Paraplagusia japonica* ^a^ (PJ)													1	33.33	1	1.56
Crustacean																
Peregrine crab *Varuna litterata*	4	7.27													4	6.25
Oriental river prawn *Macrobrachium nipponense* (MN)	2	3.64			2	3.64									4	6.25
Kuruma prawn *Marsupenaeus japonicus* (MJ)			1	1.82											1	1.56
Reptile																
Chinese water snake *Enhydris chinensis*									2	33.33					2	3.13
Aves																
Little grebe *Tachybaptus ruficollis*	1	1.82			2	3.64									3	4.69
**b. Nonfood species**																
Pisces																
Mosquitofish Gambusia affinis			1	1.82	1	1.82									2	3.13
Chulae's goby *Mugilogobius chulae*			1	1.82											1	1.56
Goby *Microphilypnus* sp.^b^											1	33.33			1	1.56
Bacteria																
*Aeromonas diversa*	1	1.82													1	1.56
Idiomarinaceae bacterium^b^									1	16.67					1	1.56
*Shewanella algae* ^a^													1	33.33	1	1.56
*Shewanella bicestrii*	3	5.45	1	1.82											4	6.25
*Shewanella decolorationis*	1	1.82													1	1.56
*Shewanella oneidensis* ^a^			1	1.82	1	1.82									2	3.13
*Shewanella* sp. 1					1	1.82									1	1.56
*Shewanella* sp. 2^b^	1	1.82													1	1.56
*Vibrio diabolicus*													1	33.33	1	1.56
*Vibrio parahaemolyticus*													1	33.33	1	1.56
Amoeba																
*Acanthamoeba* sp.^a^									1	16.67					1	1.56
Rotifer																
*Brachionus* sp.			1	1.82											1	1.56
*Philodina megalotrocha* ^b^			1	1.82											1	1.56
*Macrotrachela quadricornifera* ^b^			1	1.82											1	1.56
Uncultured bdelloid rotifer^b^			1	1.82											1	1.56
Diatom																
*Cyclotella cryptica* ^a^	1	1.82													1	1.56
Amphipoda																
*Awacaris yezoensis* ^a^													1	33.33	1	1.56
Insect																
Ant *Pheidole megacephala*											1	33.33			1	1.56
Moth *Garrha* sp.^a^													1	33.33	1	1.56
Mosquito *Cladotanytarsus gracilistylus*					2	3.64									2	3.13
Mammal																
Eurasian otter *Lutra lutra*	1	1.82	7	12.73	7	12.73	2	33.33							17	26.56

Note

Numbers in parentheses following “Habitat type” and “Total” indicate the total sample size for each spraint sample. Codes in parentheses following some species names show the abbreviations that refer to Table 1. %: frequency of all samples in the habitat type.

^a^ The highest similarity of sequence and compared data is higher 90% but less than 98%.

^b^ Higher 80% but less than 90%. Species without superscript a or b indicate that the similarity is higher than 98%.

The second group comprised “nonfood species,” including bacteria, amoeba, diatoms, small insects (e.g., ants and mosquitos), and small invertebrates, as well as the otters’ sequences themselves. A total of 59 sequence records (38.06% of all successful sequences) were identified as nonfood species, including 18 bacteria (11.61%), 2 unicellular organisms (amoeba and diatom, 1.29%), 4 rotifers (2.58%), 1 amphipoda (0.65%), 4 insects (2.58%), and 26 Eurasian otter sequences (16.77%).

Further, three ambiguous food species, the small mosquito fish *Gambusia affinis* (Baird & Girard, 1853), and two small gobies *Mugilogobius chulae* (Smith, 1932) and *Microphilypnus* sp. had been considered as “nonfood species” (4 sequences, 2.58% of all successful sequences) for their tiny size and coexistence with potential predators as the food species in the same fecal samples (see Section 4). They also were considered as “indirect food species,” that is, not hunted by otters for food. All prey and nonfood species are listed in Table 3b. In this study, no mammal species were sequenced, aside from the Eurasian otter itself. *Lutra lutra* were found in 17 spraint samples in 10 collection sites with 26 sequence reads. I considered these to be the mtDNA COI/ COIII gene sequences of the donor of the spraint and catalogued them as “nonfood species.”

Finally, a third type of sequence was observed. Two successfully sequenced COI segments from selected PCR products (409 and 200 bp in length, respectively) had no similar sequences in either BOLD or GenBank. I maintained these in the list of sequencing results as both were sequenced at high quality.

### 3.2 Prey species number in spraint samples

**FIGURE 5:**
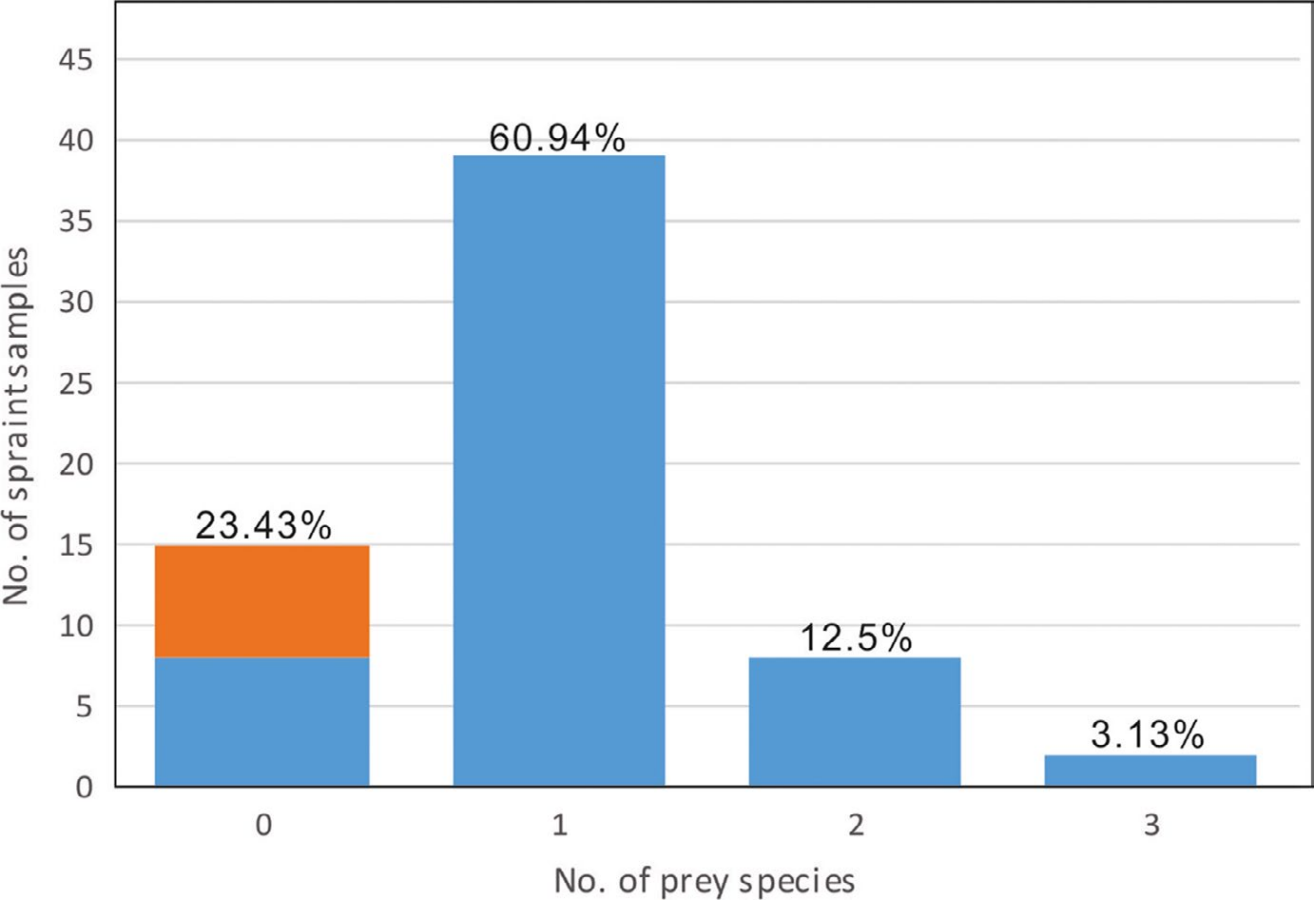
Number of prey species detected in spraint samples. Spraints containing only a single prey were most frequent, *that is,* up to 39 samples among the 64 tested. The orange color indicates 7 spraints containing nonfood species but prey

In total, 39 spraint samples contained 1 prey species, which is 60.94% of all 64 tested samples. Eight samples contained 2 species (12.5%), and only 2 samples contained 3 prey species (3.13%). There were 15 samples with no food species inside. However, half of them were host valid species sequences, which were identified as nonfood species (from 1 to 4 species). Only 8 samples hosted no valid sequence, given the absence of PCR products using any of the 9 primer sets, or poor sequencing results in some PCR products, despite them passing the electrophoresis check and being selected for sequencing (Figure 5).

### 3.3 PCR success ratio of each primer sets

Among the 9 primer sets, the numbers of successful PCR procedures and sequences ranged from 4 to 36 (Table 4), which means the success rates of each primer set ranged from 6.25% to 56.25% (assuming all 64 spraint samples contained the groups that each primer set targets). The FishF2/R2 showed the most successful rates and identified one avian sequence, 21 fish sequences, 4 bacteria sequences, and 10 Eurasian otter sequences, in which 21 of them belong to target food species. Moreover, the Til9020F/RM and FishF1/R1 showed good results with 30 (46.88% successful rate) and 22 (34.38%) sequences with fine resolution. The former one was designed for the CO III gene segment of the tilapia species complex, but also works on other food species, including 1 other fish *Chelon affinis* (up to 96.46% in similarity), 1 shrimp *Marsupenaeus japonicas* (98.82%), and 2 Eurasian otter sequences (100%).

**TABLE 4:** Summary of taxa numbers sequenced by nine primer sets

Primer sets	Readable sequence no.	Food species	Nonfood species	No match	Aves	Pisces	Snake	Crustacean	Insect	Rotifer	Amphipoda	Unicellular	Bacteria	Otter
I (BirdF1/RM)	4	1	3	–	–	1	–	–	–	–	–	–	–	3
II (VF1/VRM)	15	10	4	1	–	6	2	2	–	–	–	–	4	–
III (chmf4/r4)	5	5	–	–	–	4	–	1	–	–	–	–	–	–
IV (FF2d/FR1d)	19	4	15	–	–	2	2	–	–	–	–	–	4	11
V (FishF1/R1)	22	15	6	1	2	12	1	1	–	–	–	–	5	–
VI (FishF2/R2)	36	21	15	–	1	21	–	–	–	–	–	–	4	10
VII (LCO1490/HCO2198)	13	6	7	–	–	–	–	6	1	3	1	1	1	–
VIII (LepF1/R1)	9	2	7	–	–	2	–	2	3	1	–	1	–	–
IX (Til9020F/RM)	30	28	2	–	–	27	–	1	–	–	–	–	–	2
Total	153	92	59	2	3	75	5	13	4	4	1	2	18	26

Crustaceans are important food resources for Eurasian otters (Heggberget & Moseid, 1994; Krawczyk et al., 2016). In this work, 13 crustacean sequences were identified successfully by LCO1490/HCO2198 (6 sequences), LepF1/R1 (2), VF1/VRM (2), chmf4/r4 (1), FishF1/R1 (1), and LepF1/R1 (1). The former two sets showed high diversity of sequenced species (with 6 and 5 catalogued groups, respectively) and had high potential to discern unexpected species (Table 4).

## 4 DISCUSSION

### 4.1 Food species identified in this study

The Eurasian otter is a generalist predator and thus displays foraging strategies as adaptive responses adjusted to food availability (Almeida et al., 2012; Barrientos et al., 2014; Krawczyk et al., 2016). The results of this work suggest that the Kinmen otters displayed clear piscivorous foraging, especially on the introduced tilapia species. While the dominance of invasive tilapia has become a great threat to local endangered fishes (Chen et al., 2013), they appear to provide plentiful food resources for predators like otters. Not surprisingly, tilapia were the most frequent food species identified in Kinmen, present in 55% of the analyzed 64 spraint samples, and in 70% of spraint samples with prey inside (*n* = 50). Two tilapia species, *Coptodon zillii* and *Oreochromis niloticus*, are consumed by otters frequently for their good body size and high density in the inland waters of Kinmen, as well as their relatively poor movement ability in shallow water. Consequently, tilapia are presently the main food species of Kinmen otters.

Otter diet reflects local availability of relevant prey species (Kruuk, 2010). Chen et al. (2013) reported that there were 68 inland water fishes recorded on Kinmen Island. Most of the native ones are small in size. The native freshwater fishes we found from spraint samples are crucial carp (in 2 spraint samples), carp (1), and blotched snakehead *Channa maculata* (1). These are the largest native freshwater fish species on Kinmen. However, in this study, a very low percentage of otters consumed the native fishes. The limited appearance of native species reflects their low population density in Kinmen, having been seriously threatened by tilapia. The body size selection, individual numbers and easy‐to‐hunt fishes occupied the bulk of Eurasian otter diets; thus, the Kinmen otters modified their trophic niche to turn to non‐native prey, similar to European populations (Barrientos et al., 2014).

Other native nonfish food species were also rarely detected in this study. It is possible that this is an effect of the small sample size (*n* = 64), or that there is simply no need for Kinmen otters to hunt alternative prey, given that tilapia constitute a sufficient food resource.

### 4.2 Nonfood species identified in this study

In total, 59 sequences were found in this work that derived from nonfood species, including 4 bacteria groups (genera *Aeromonas*, *Vibrio*, *Shewanella* and one similar to family Idiomarinaceae with 80.86% similarity; 18 sequences), 1 amoeba (1), 1 amphipoda (1), 1 diatom (1), 3 fishes (4), 3 insects (4), some rotifers (4), and Eurasian otter (26). Among these records, the invertebrate species are too small to be assumed without doubt as otter prey. Aged spraints have more potential exposure to external contaminants, as reflected in the nonfood DNA sequences recovered (McInnes et al., 2017), such as microbes, fungi, ants, and coprophagous invertebrates (e.g., flies and their eggs). Besides, King et al. (2008) suggested that fecal matter may be contaminated with planktonic organisms while collected in the sea and potentially even in fresh water. In the case of otters, contamination with water is inevitable, as they typically defecate soon after leaving the water, and water from their bodies wets the spraints. Tiny aquatic creatures such as rotifers and diatoms were similarly found in this study (Table 3b).

Secondary predation may also be evident in otter diets (Kumari et al., 2019). Some organisms were probably incidentally consumed by larger organisms predated by the otters. For example, the Kinmen otters feed on tilapia, euryphagous fishes, which in turn feed on smaller, dead organisms, or even the organic matter of other animals (e.g., feces) that are thus later present in the otters’ guts. Here, I have the opportunity to check the relationships between the predator (the donor of the spraint) and potential “indirect food species,” given that I list the diet analysis results of each spraint sample one by one. In doing so, I suggest that some tiny fishes such as mosquito fish and small size gobies should be considered as indirect food species, as they were observed alongside their possible predators, the little grebe, Nile tilapia *Oreochromis niloticus* and Japanese tiger prawn in this work (see samples #4, #8, #20, #63 in Table 3). I also suggest that organisms unintentionally ingested by means of being attached to larger organisms (e.g., parasites) predated by the otters should be considered as nonfood or indirect food species.

There were 26 valid sequences of *Lutra lutra*, comprising 16.77% of all successful sequences among this work. When barcoding with mtDNA sequences, it is difficult to distinguish whether these otter DNA fragments were from donor or prey species, that is, if cannibalism occurred. In the absence of any evidence suggesting cannibalism, I consider these to be the DNA sequences of spraint donors and thus did not include the in the diet species list.

### 4.3 The quality of analyzed spraint samples

Sample freshness is very important in molecular scatology studies. In this study, I used the highest quality spraint‐derived DNA as possible, having prescreened the extracts with a panel of microsatellites (see Hung et al., 2004; Park & Cho, 2017). Such genotypes could be used in a later study to connect individual identifications to diet information, if needed.

In general, only the single‐band PCR products were selected for sequencing, except for those amplified with the Til9020F/RM primer set. Most of the PCR products amplified with these primers showed multiple bands (e.g., Figure 3d), but they could always be sequenced successfully and with good quality (sequencing using the forward primer Til9020F only). It is interesting that the Til9020F/RM primer set worked very well in this work by detecting prey successfully in 29 spraint samples, not only on tilapia (27 samples) but also on other fish (1) and shrimp (1) species. The success of this primer set can be attributed to its custom design to target tilapia specifically, on the understanding that these species are abundant in Kinmen and probably constitute prey. Reviewing potential prey species, and designing appropriate custom primers, is therefore recommended in future studies where possible prey can be identified. This improves on the use of published universal primers, which are better used to “discovery” novel prey than to amplify known prey species.

Fifteen spraint samples showed the absence of food species inside. It is possible that they did contain nonfood species, and that they were false negative results. The production of such false negatives (i.e., failure of amplification when target food DNA is or was present in the sample) could be due to degradation of the DNA present in the sample, failure of the DNA extraction, or failure of the PCR amplification (Deagle et al., 2005; Kalle et al., 2014; King et al., 2008). In studies where the real diet is unknown, such as in this study, which focused on wild individuals, monitoring the incidence of false negatives is extremely difficult and their complete elimination is unlikely to be possible. My solution was to collect spraint samples as fresh as possible for DNA extraction, to maximally reduce DNA degradation.

For adult Eurasian otters, defecation is also an important behavior to mark their territories. Beside food remains, spraints include the fairly inconspicuous secretions of two anal glands, plus a jelly‐like substance secreted somewhere in the intestine itself. Occasionally, a spraint consists of nothing but this jelly. When Eurasian otters produce this jelly spraint, the defecation is not for purposes of elimination, but for scent communication (Kruuk, 2010). Among the nonfood species spraints, 7 samples were noted as jelly or mucus samples (#17, #39, #44, #45, #47, #60 and #64), which is probably the reason they contained no food materials. However, it is not inevitable that jelly or mucus will contain no diet information. In other jelly or mucus samples (#19 and #43), I detected tilapia sequences, though no scales or spiny bones were observed. Aside from jelly or mucus, 5 spraints were greenish, small in size, soft and wet, and no scales or bones (or very few) were found inside (#7, #21, #31, #32, and #38). Such spraints were considered to be feces belonging to unweaned cubs and were easily distinguished from their mother's (or other adults’) spraints. When the cubs are small and feeding only by suckling inside the natal holt, their mother typically eats their spraints. Later, during the days of the cubs’ life outside the natal holt, when they are about 2 months old and before weaning, it is possible for the cubs to leave spraints beside their mother's ones outside the natal holt. Occasionally, such spraints comprised no food species DNA.

**FIGURE 6:**
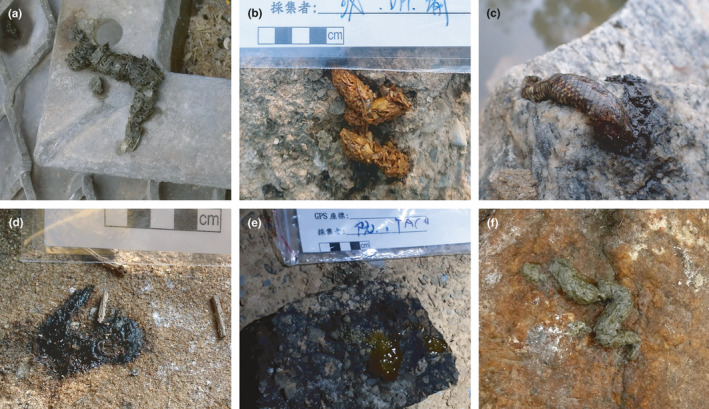
Spraint samples of the various forms and conditions produced by Eurasian otters on Kinmen Island. (a) A standard‐looking spraint collected in Tai Lake, Tai‐140 (sample #28 in Table 1); (b) a spraint containing crab remains collected in Guangqian River, GQR‐37 (#54); (c) a spraint covered by snakeskin collected in Ci lake, Ci‐38 (#50); (d) a mucus sample collected in Mintan Lake, MT‐16 (#39); (e) a jelly‐like sample collected in Qionglin Reservoir, QL‐41 (#45); (f) a soft, small spraint with few spiny bones collected in Shanwai Stream, YB‐72 (#37)

In two of the spraint samples, no food species were detected, but scales and spiny bones of fish were visually apparent inside them (#36 and #37; see Figure 6). Both spraint samples were very small, but had passed the prescreening test and could even be identified at the individual level. In these cases, an abundance of nonfood species’ DNA may have caused their preferential amplification in the PCR.

### 4.4 A recommended modified procedure

It is better to have an idea of the possible prey fauna in advance, and to estimate the most abundant prey species and their species diversity. Based on this information, designing customized species‐specialized supplementary primers—in place of sole reliance on published universal barcoding primers—would likely be very helpful. However, given the variation observed in the success rates of each primer set in this study, it is evident that some primer sets did not work so well, and could be replaced by others. For example, the BirdF1/RM set was intended to amplify COI sequences of avian prey, but only worked on 4 spraint samples (#2, #5, #20, #39) in this study. Three resulting sequences were otter COI segments and the remainder were Nile tilapia, not birds. Such a primer set might therefore be considered as supplementary and used only when the spraint contains evidence of feather or crustacean appendages. All this considered, I propose the following recommendations for modifying my procedures when applying these to future Kinmen otter diet studies:

1. Execute PCR amplification with 5 primer sets: VF1/VRM, FishF1/R1, FishF2/R2, LepF1/R1 (or LCO1490/HCO2198) and Til9020F/RM
2. Check for visible hard components of prey in the spraint sample. Select additional corresponding primer sets for the observed prey groups (i.e., when seeing feathers, use BirdF1/RM; for frog bones, use chmf4/r4).
3. When checking the PCR products
  3a. If no positive PCR products are observed among the 5 or more reactions, record the sample as producing no data, that is, no prey species inside. End the use of this sample.
  3b. If some PCR products show clear bands, select the successful single‐band PCR products (or multiple clear bands with the Til9020F/MR primer set) for sequencing. Identify the sequences uses GenBank or BOLD.
  4a. If more than half the sequencing results (>3) refer to one single prey species, note the result and end the experiment.
  4b. If two or more species were identified, or multiple bands occurred in any PCR products but no valid sequence was produced, execute PCRs with the remaining primer sets (i.e., up to 9 PCRs in total).

In the first step, the first four recommended primer sets are the most universal in barcoding analysis, and can amplify most animals’ COI genes assuming they are not degraded. When some bands are observed in the step but prove difficult to Sanger sequence, I suggest running all primer sets before noting that the sample produced no sequencing data. Repeated PCR tests provide several opportunities to detect the diet species in a spraint sample; in this case, such efforts did yield data eventually.

This approach cannot be applied in species that consume many prey individuals each day (such as middle and large size carnivores and piscivores, especially those that feed on countless small invertebrates in a day), as they are likely to have too many prey species in their scats. The sDNA metabarcoding methods are much more suitable in such cases if funds permit their use. Those with few prey individuals/species are otherwise ideal subjects for the series of Sanger‐sequencing reactions proposed herein.

## 5 CONCLUSIONS

Given that Eurasian otters generally consume a small number of prey species, this procedure is ideal for discerning those apparent in the spraints of specific individuals or populations. By using common PCR and Sanger‐sequencing based approaches, samples can be analyzed on an individual basis at low cost and with limited technical expertise. By maintaining detailed records in the field, diet data can be connected with other information, for example, locality, habitat conditions, timing, spraint morphology, and to the individual identities of otters. The collection and analysis of such data in combination can yield plentiful data for understanding dietary ecology.

## CONFLICT OF INTEREST

The author declares no conflicts of interest.

## AUTHOR CONTRIBUTION

**Nian‐Hong Jang‐Liaw:** Conceptualization (lead); Data curation (lead); Formal analysis (lead); Funding acquisition (lead); Investigation (lead); Methodology (lead); Resources (lead); Validation (lead); Visualization (lead); Writing‐original draft (lead); Writing‐review & editing (lead).

## Supporting information

Appendix S1

Appendix S2

Appendix S3

## Data Availability

Data described herein are detailed in Table 1. Appendix S2 provides descriptions of all readable sequences, including sample information and identification (available from Dryad, https://doi.org/10.5061/dryad.msbcc2fz2). Final DNA sequence assemblies are uploaded as Appendix S3 (available from Dryad, https://doi.org/10.5061/dryad.msbcc2fz2). All data are available from the author.
