# Correction to “A Barcoding‐Based Scat‐Analysis Assessment of Eurasian Otter *Lutra lutra* Diet on Kinmen Island”

**DOI:** 10.1002/ece3.73144

**Published:** 2026-02-19

Jang‐Liaw, N.‐H. A Barcoding‐Based Scat‐Analysis Assessment of Eurasian Otter *Lutra lutra* Diet on Kinmen Island. *Ecology and Evolution*, 2021; 11: 8795–8813. https://doi.org/10.1002/ece3.7712

In Table 2 of the originally published article, the naming of the COI primer sequences FishR1 and FishF2 was incorrectly swapped for Set V and Set VI.

**TABLE 2:** PCR primer sets or cocktails used to amplify COI/COIII segments in this study.

Set	Primer name/Cocktail name	Sequence 5′–3′	Target gene	Target animals	Approx. sequence length (bp)	References
I	BirdF1	TTCTCCAACCACAAAGACATTGGCAC	COI	Birds	650	Hebert et al. (2004a)
	BirdRM (mixed with BirdR1, R2 and R3)					
	BirdR1	ACGTGGGAGATAATTCCAAATCCTG				Hebert et al. (2004a)
	BirdR2	ACTACATGTGAGATGATTCCGAATCCAG				Hebert et al. (2004a)
	BirdR3	AGGAGTTTGCTAGTACGATGCC				Hebert et al. (2004a)
II	VFM		COI	Mammals, reptiles, fish, amphibians, and some insects	650	
	VF1	TTCTCAACCAACCACAAAGACATTGG				Ivanova, Dewaard and Hebert (2007)

	VRM (mixed with VR1 and VR1d)					
	VR1 (FishR1)	TAGACTTCTGGGTGGCCAAAGAATCA				Ivanova, Dewaard and Hebert (2007)
VR1d	TAGACTTCTGGGTGGCCRAARAAYCA				Ivanova, Dewaard and Hebert (2007)
III	chmf4	TYTCWACWAAYCAYAAAGAYATCGG	COI	Amphibians	650	Che et al. (2014)
	chmr4	ACYTCRGGRTGRCCRAARAATCA				Che et al. (2014)
IV	FF2d	TTCTCCACCAACCACAARGAYATYGG	COI	Fishes	650	Ivanova et al. (2007)
	FR1d	CACCTCAGGGTGTCCGAARAAYCARAA				Ivanova et al. (2007)
V	FishF1	TCAACCAACCACAAAGACATTGGCAC	COI	Fishes	650	Ward et al. (2005)
	FishR1	**TAGACTTCTGGGTGGCCAAAGAATCA**				Ward et al. (2005)
VI	FishF2	**TCGACTAATCATAAAGATATCGGCAC**	COI	Fishes	650	Ward et al. (2005)
	FishR2	ACTTCAGGGTGACCGAAGAATCAGAA				Ward et al. (2005)
VII	LCO1490	GGTCAACAAATCATAAAGATATTGG	COI	Various phyla from the animal kingdom	650	Folmer et al. (1994)
	HCO2198	TAAACTTCAGGGTGACCAAAAAATCA				Folmer et al. (1994)
VIII	LepF1	ATTCAACCAATCATAAAGATAT	COI	Lepidoptera	650	Hebert et al. (2004b)
	LepR1	TAAACTTCTGGATGTCCAAAAA				Hebert et al. (2004b)
IX	Til9020F	TAACAATRTACCAATGATGACGAG	COIII	Tilapia	See below	This study
	TilMR (mixed with Mos9516R, Nil9464R, Esc9305R and Zil9212R)					
	Mos9516R	ACCCAAAGTGATGTTCTGATG			548	This study
	Nil9464R	GCAGACGGCCAGGAAAGTAGAGC			500	This study
	Esc9305R	ATAGTTAGGGCGAGGGATTGAA			346	This study
	Zil9212R	AAGACGGCGGTGTTAAGCAGAGG			254	This study

The sequence TCGACTAATCATAAAGATATCGGCAC, listed as FishR1 in Set V, is in fact the forward primer FishF2. Conversely, the sequence TAGACTTCTGGGTGGCCAAAGAATCA, listed as FishF2 in Set VI, is the reverse primer FishR1. The correct primer sequences are provided in the updated Table 2 below.

This correction pertains solely to the labeling of primer names in Table 2. The primer sequences themselves, laboratory procedures, and all analyses and conclusions presented in the article remain unchanged and are not affected by this error.

I apologize for this error.

