# Supplementary material for: A barcoding‐based scat‐analysis assessment of Eurasian otter Lutra lutra diet on Kinmen Island
Source: Ecol Evol. 2021 Jun 4;11(13):8795–813. doi: 10.1002/ece3.7712 (PMC8258194; doi:10.1002/ece3.7712)
Supplement: Supplementary file 1 — Appendix S1 [file ECE3-11-8795-s001.docx]

**Appendix S1.**

**A modified DNA extraction procedure used in this work**

(Using DNeasy Blood & Tissue Extraction Kit)

1. Remove fishbone, scales and other solid objects from sprain sample.
2. Transfer 0.2g/200ul mud-like faeces sample into a 2ml centrifuge tube. Remove the ethanol buffer and dry the sample before next step.
3. Add 360 µl ATL buffer and 40 µl proteinase K into sample.
4. Vibration. Dry bath in 56℃ overnight.
5. Add 400 µl AL buffer and dry bath for another 10 minutes.
6. Add 400 µl 99.5% Ethanol. Shake the mixed buffer.
7. Transfer the mixed buffer into a spin column provided by kit. Spin in 9500 rpm for 1 minutes. Abundant the separate out solution.
8. Add 500 µl AW1 buffer. Spin in 9000 rpm for 1 minutes. Abundant the separate out solution.
9. Add 500 µl AW2 and spin with 14000 rpm for 3 minutes. Abundant the separate out solution.
10. Spin column is transfer to a new centrifuge tube, and 80 µl AE buffer is added with heat to 65 ℃.
11. Spin the sample with 9000 rpm for 2 minutes. Keep the separate out solution (extracted DNA) carefully.

Appendix S1. A modified DNA extraction procedure used in this work (available from Dryad, doi.org/10.5061/dryad.fn2z34ttj).

Appendix S2. Information on the 153 readable DNA sequences produced in this study (available from Dryad, doi.org/10.5061/dryad.ht76hdrfm). The original sequence data is reported in S3.

Appendix S3. The 153 readable DNA sequences in this study (available from Dryad, doi.org/10.5061/dryad.msbcc2fz2).
